# Vector Auto-Regressive Deep Neural Network: A Data-Driven Deep Learning-Based Directed Functional Connectivity Estimation Toolbox

**DOI:** 10.3389/fnins.2021.764796

**Published:** 2021-11-23

**Authors:** Takuto Okuno, Alexander Woodward

**Affiliations:** Connectome Analysis Unit, RIKEN Center for Brain Science, Wako, Japan

**Keywords:** vector auto-regressive deep neural network, directed functional connectivity, fMRI, Granger causality, Alzheimer’s disease

## Abstract

An important goal in neuroscience is to elucidate the causal relationships between the brain’s different regions. This can help reveal the brain’s functional circuitry and diagnose lesions. Currently there are a lack of approaches to functional connectome estimation that leverage the state-of-the-art in deep learning architectures and training methodologies. Therefore, we propose a new framework based on a vector auto-regressive deep neural network (VARDNN) architecture. Our approach consists of a set of nodes, each with a deep neural network structure. These nodes can be mapped to any spatial sub-division based on the data to be analyzed, such as anatomical brain regions from which representative neural signals can be obtained. VARDNN learns to reproduce experimental time series data using modern deep learning training techniques. Based on this, we developed two novel directed functional connectivity (dFC) measures, namely VARDNN-DI and VARDNN-GC. We evaluated our measures against a number of existing functional connectome estimation measures, such as partial correlation and multivariate Granger causality combined with large dimensionality counter-measure techniques. Our measures outperformed them across various types of ground truth data, especially as the number of nodes increased. We applied VARDNN to fMRI data to compare the dFC between 41 healthy control vs. 32 Alzheimer’s disease subjects. Our VARDNN-DI measure detected lesioned regions consistent with previous studies and separated the two groups well in a subject-wise evaluation framework. Summarily, the VARDNN framework has powerful capabilities for whole brain dFC estimation. We have implemented VARDNN as an open-source toolbox that can be freely downloaded for researchers who wish to carry out functional connectome analysis on their own data.

## Introduction

There is a rich history of structural and functional connectome analysis of neuroimaging data of the whole brain, such as those acquired from MRI (magnetic resonance imaging). One important goal is to study the causal relationship between activity in different brain regions and the brain’s functional circuitry. The estimation of the functional connectome is an important step for understanding brain function (Gao et al., 2011; Krueger et al., 2011; Hahn et al., 2019) and for the diagnosis of brain lesions (Dauwels et al., 2010). In the literature, there is a notable lack of data-driven approaches to functional connectome estimation that leverage the advantages of the current state-of-the-art deep neural network architectures and training methodologies. Such approaches should have an advantage in terms of representational power and trainability. Some recent examples of neural network-based measures of GC have been proposed, such as neural network GC (NN-GC) (Montalto et al., 2015), RNN-GC (Wang et al., 2018), Echo State Network GC (ES-GC) (Duggento et al., 2019), and DNN-GC (Chivukula et al., 2018). Accordingly, we propose a new approach for whole-brain analytics based on a *vector auto-regressive deep neural network* (VARDNN) architecture that can deal with a large number of time series. The model comprises a set of nodes with a deep neural network (DNN) structure. Each node maps with arbitrary divisions of space, such as an anatomical brain region from where a representative neural signal (time-series) can be estimated. At each time step, a node outputs a signal by considering exogenous signals into the system, signals from all other nodes, and feedback from the node back to itself. The model can be trained on time series data of brain activities and recordings of arbitrary exogenous inputs. Functional connectome measures can be derived from the model after the training process. A wide variety of data can be used for training, such as fMRI BOLD or calcium imaging data; our approach is fully data-driven. We seek a non-parametric model that not only fits the data but also describes important dynamical properties and can complement hypothesis-driven approaches (Valdes-Sosa et al., 2011). We defined two types of *directed functional connectivity* (dFC) measures based on the VARDNN, namely VARDNN-DI (Directional Influence, our new measure) and VARDNN-GC (based on Granger causality).

Many types of functional connectome analysis algorithms are currently used. *Functional connectivity* (FC), which is simply calculated by the correlation coefficient of *pairwise* time-series, is the most basic measure to analyze brain region relationships. This term was originally defined as the temporal correlation of a neurophysiological index measured in different brain areas (Gerstein et al., 1989; Friston et al., 1993). *Partial Correlation* (PC) is a *conditional* version of correlation. This is calculated by the correlation of regression residuals with other time-series. When two factors show a strong correlation, it may be necessary to consider the presence of a third factor. PC can remove this kind of third factor effect and show a normalized correlation. FC based on both correlation and partial correlation only consider the *zero-lag* relationship between signals. On the other hand, *Granger causality* (GC) is based on a vector auto-regression (VAR) scheme and is categorized as a *predictive* type measure. The original formulation of GC was for the *pairwise* case but has since been extended to the *conditional* and *multivariate* cases (Granger, 1969; Geweke, 1982; Goebel et al., 2003). Furthermore, an information-theoretic approach to understanding causal relationships, called *transfer entropy* (TE), has been developed (Schreiber, 2000). This has been shown to be equivalent to GC when the time series dynamics under study are normally distributed (Barnett et al., 2009). GC and TE are directional, thus these measures are categorized as *directed functional connectivity* (dFC) (Friston et al., 2013), while FC and PC are categorized as *undirected functional connectivity*.

Some measures depend on a linear regression scheme, thus their coefficients and residuals are calculated by ordinary least squares (OLS). But OLS has several drawbacks: if the predictor variables (P) are larger than the observed data (N), the solution faces overfitting (*P* >> *N* problem). Furthermore, if the observed data are strongly correlated with each other, the Gram matrix cannot be inverted and OLS cannot calculate the correct solution (multicollinearity problem). In the case of analyzing functional MRI data, sometimes several regions have strong correlations, or the observed data may be smaller than the region number. Here, functional connectome analysis algorithms face several OLS problems. To avoid these situations, countermeasure techniques can be applied. For example, PC has been combined with a L1-norm regularized regression scheme (SPC-EN) (Ryali et al., 2012), and multivariate GC has been combined with Principal Component Analysis (PCA-cGCM) (Zhou et al., 2009) to reduce the data dimension and transform the data into an orthogonal vector space.

Accurate estimation of the brain’s functional connectome is an important topic for neuroscience. One challenge is that the estimated functional connectome values are not consistent across analysis algorithms, which can cause confusion when interpreting the results of a particular measure. Dauwels et al. (2010) attempted to systematically investigate EEG (electroencephalogram) synchrony with a special focus on the early diagnosis of Alzheimer’s disease (AD). They evaluated 29 types of functional measures, including the correlation coefficient and related measures, phase synchrony indices, GC, and stochastic event synchrony (SES) (Dauwels et al., 2008). Results showed that only two measures, GC and SES, were able to convincingly distinguish MCI (mild cognitive impairment) patients from age-matched controls. Another investigation was performed by Smith et al. (2011). They evaluated 20 functional methods, including the correlation coefficient and related measures, mutual information (Shannon, 1948), GC, the LiNGAM algorithm (Shimizu et al., 2006), and several Bayes Net modeling algorithms. Twenty-eight types of synthetic fMRI BOLD (blood oxygen level dependent) signals were generated by the DCM model (Friston et al., 2003), and all methods were evaluated for their estimation of the ground truth connectivity. In the results, the PC regularized inverse covariance estimation, and several Bayes Net methods showed the highest sensitivity. Prando et al. (2020) also evaluated the performance of functional connectome estimation using a number of methods. They compared sparse vs. spectral DCM, multivariate GC, LiNGAM, and several Bayes Net modeling algorithms. In total, 9 methods were evaluated using synthetic fMRI BOLD signals generated by the DCM model. The results showed that the sparse DCM method gave the highest accuracy and multivariate GC was competitive. Overall, Granger causality-based measures appear to give consistent performance for functional connectome estimation from both empirical and synthetic signals. A purely data-driven approach makes less assumptions about the underlying system generating the dynamics; hence, it can be applied more broadly to different types of phenomena and is less computationally expensive.

To evaluate our VARDNN approach we tested the performance of its dFC measures against 14 other analysis algorithms, including *zero-lag* and *predictive* types, and *pairwise* and *multivariate* strategies. Included in these algorithms, countermeasure techniques, such as PCA, Elastic Net (Zou and Hastie, 2005) and Partial Least Squares (PLS) (Wold et al., 2001) were combined with PC and multivariate GC. All measures were evaluated on biologically plausible synthetic signals generated by DCM and the reduced Wong-Wang neural mass model (Wong and Wang, 2006; Deco et al., 2013). In particular, we found that both measures, especially VARDNN-DI, were able to consistently provide competitive and top-ranking performance, especially when the size of the number of nodes increased. Furthermore, we evaluated VARDNN performance by analyzing empirical fMRI BOLD data. Healthy control and Alzheimer’s disease fMRI BOLD data were acquired from the *Alzheimer’s Disease Neuroimaging Initiative* (ADNI2) database, and functional connectomes were calculated by each of the analytical methods studied. Functional connectome values were then used to compare group separation ability of Alzheimer’s disease within a subject-wise evaluation framework. Functional connectomes estimated by the VARDNN measure was able to capture the causal relationship between brain regions and showed a significant difference between healthy control and Alzheimer’s disease data.

## Materials and Methods

### Vector Auto-Regressive Deep Neural Network Architecture

**FIGURE 1:**
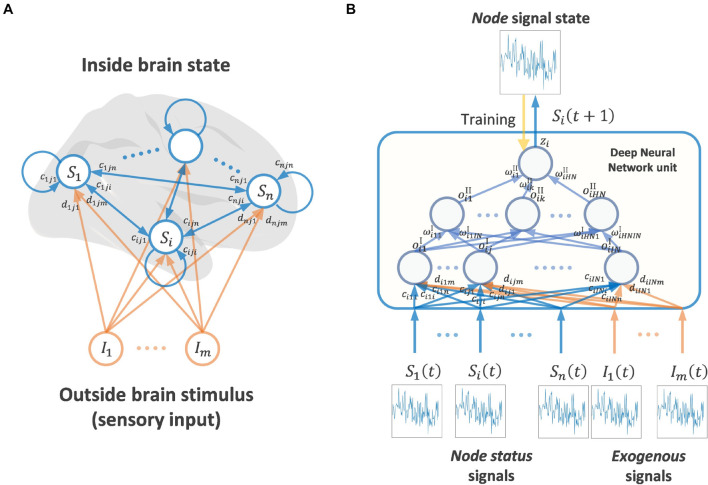
**(A)** Whole brain node state network and stimulus coming from outside brain (mainly sensory input) **(B)** Showing signaling framework of one node state. A Deep Neural Network node receives the signal state of other brain nodes and exogenous signals (outside brain stimulus). A DNN unit calculates one output signal, in this case the next time step of the signal state of a node. The DNN unit is trained by teacher signals and the input of other node states and exogenous signals.

Figure 1 shows a schematic diagram of the VARDNN framework. The architecture consists of a set of nodes that can be assigned to different regions of the brain from which experimental signals are recorded. The node state vector is ***S***(*t*) = (*S*_1_(*t*), ⋯, *S*_*n*_(*t*)), where *S*_*i*_(*t*) ∈ ***R***, *i = 1,..,n*, represents the *i*th node’s state at discrete time *t* = 1,2,…. All nodes are part of a fully connected network structure, receiving input from every other node. Additionally, each node receives exogenous signals from outside of the brain, *I*_*q*_(*t*) ∈ ***R***, *q = 1,…,m*, expressed as an exogenous signal vector ***I***(*t*) = (*I*_1_(*t*), ⋯, *I*_*m*_(*t*)). These signals can arbitrarily represent any external data derived from an experimental paradigm that would be received as sensory information by the subject. All nodes contain a DNN unit with trainable weights across two hidden layers. For a node *i*, *c*_*i*,*j*,*p*_, *d*_*i*,*j*,*q*_ ∈ ***R*** are the connection weights between its first hidden layer neurons *x*_*i,j*_, *j = 1,…,IN*, and the input from every other deep neural network unit node *p = 1,…,n* (including its self-connection), or from the exogenous signal inputs (*q = 1,…,m*), respectively. At each time step, signals pass between nodes and are processed by the DNN units. Within a DNN, the output of the first hidden layer for node *i* is expressed as


(1) $$O_{i}^{I}\;=\;{g(C_{i}{S\left(t\right)}^{T}\;+\;D_{i}{I\left(t\right)}^{T}\;+\;{B_{i}}^{T})}^{T}\;$$

where


$$\begin{array}{c} C_{i}=\left(\begin{array}{ccc} c_{i,1,1} & \cdots & c_{i,1,n}\\ \vdots & \ddots & \vdots \\ c_{i,IN,1} & \cdots & c_{i,IN,n} \end{array}\right),D_{i}=\left(\begin{array}{ccc} d_{i,1,1} & \cdots & d_{i,1,m}\\ \vdots & \ddots & \vdots \\ d_{i,IN,m} & \cdots & d_{i,IN,m} \end{array}\right),\\ B_{i}=\left(b_{i,1},\cdots ,b_{i,IN}\right) \end{array}$$

where *b*_*i*,*j*_ ∈ ***R*** are the bias values on the first hidden layer neurons and *g*(*x*) = max(0, *x*) is the non-linear ReLU activation function (Nair and Hinton, 2010; Glorot et al., 2011). $O_{i}^{\text{I}}$ is passed to the second hidden layer neurons of node *i*, and its output is expressed as


(2) $${\text{O}}_{i}^{II}={g({\text{W}}_{i}^{I}{{\text{O}}_{i}^{I}}^{T}+{\beta_{i}^{I}}^{T})}^{T}$$

where


$$\begin{array}{r} W_{i}^{I}=\left(\begin{array}{ccc} \omega_{i,1,1}^{I} & \cdots & \omega_{i,1,IN}^{I}\\ \vdots & \ddots & \vdots \\ \omega_{i,HN,1}^{I} & \cdots & \omega_{i,HN,IN}^{I} \end{array}\right),\beta_{i}^{I}=(\beta_{i,1}^{I},\cdots ,\beta_{i,HN}^{I}) \end{array}$$

$\omega_{i,j,k}^{\text{II}}\in R$ are the weight values of the second hidden layer neurons *k = 1,…,HN*, and $\beta_{i,k}^{\text{II}}\in R$ are the biases. Finally, node *i*’s DNN second hidden layer outputs are processed by an output layer neuron:


(3) $$\begin{array}{r} z_{i}\left(t\right)=W_{i}^{II}{O_{i}^{II}}^{T}+\beta_{i}^{II} \end{array}$$

where $W_{i}^{\text{II}}=\left(\omega_{i,1}^{\text{II}},\cdots ,\omega_{i,HN}^{\text{II}}\right)$ is the output neuron’s weight vector, and $\beta_{i}^{\text{II}}\in R$ is its bias. Finally, the VARDNN’s new state at *t+1* for all nodes is taken as the value of each node’s output:


$$\begin{array}{r} S\left(t+1\right)=Z\left(t\right)=\left(z_{1}\left(t\right),\cdots ,z_{n}\left(t\right)\right). \end{array}$$

For our current implementation, we defined 2 hidden layers for each DNN unit in the VARDNN architecture. However, the DNN unit is not limited to 2 hidden layers, and other forms of DNNs can also be used in the framework. Our decision was based on this work’s focus on analyzing whole brain fMRI BOLD type data, and 2 layers were empirically determined (see Supplementary Figure 1 for a description of the procedure). Although neural networks with single hidden layers are known as universal approximators (Cybenko, 1989; Hornik, 1989), a single hidden layer requires an exponential increase in the number of hidden neurons proportional to the problem size (Goodfellow et al., 2016; Liang and Srikant, 2016). Theoretically and experimentally, deep hidden layers with smaller numbers of neurons have shown higher accuracy than shallow (single) hidden layer networks (Delalleauand Bengio, 2011; Goodfellow et al., 2014).

VARDNN can learn to reproduce specific time series data through training on teacher data. The teacher data can be defined as Teach(t)=(S′(t)1,⋯,S′n(t),I1′(t),⋯,Im′(t)), where $S\prime {}_{i}$ and $I\prime {}_{i}$ are the experimentally obtained signals of “neural activity” and (if any) exogenous inputs, respectively, for each node. Here, *t* = 1,2,…,L, where L is the number of *frames* of data at a sampling period TR. The number of nodes, *n*, corresponds to the number of input time series. This number is arbitrary and should reflect some spatial sub-division of the brain, based on the nature of the teacher data. For example, if fMRI BOLD data is used, then one can average the voxel values within each anatomical region to generate a set of representative teacher signals.

Each node is independently trained with the aim to iteratively reduce the error between the VARDNN-generated *t+1* state and the teacher data at time t+1, when the current state is set by the time *t* teacher data. With teacher data of length *L* frames, the training set for a node *i* comprises {***ϕ***_*i*_(1),…,***ϕ***_*i*_(*L*−1)}, where $\phi_{i}\left(k\right)=\left\{\mathrm{Teach}\left(k\right),S_{i}^{\prime }\left(k+1\right)\right\}$ is an input/target pair, *k* = 1,2,…,L-1. Even though these pairs are derived from time series data, they can be used independently. Thus, the shuffling and minibatch techniques can be applied in each training epoch. This feature of VARDNN is clearly different from recurrent neural network (RNN) architectures as VARDNN is simply trained by input/target patterns but not sequences.

Within each training epoch, for each training pair ***ϕ***_*i*_(*k*), the VARDNN node state and exogenous input are set to ***Teach***(*k*), and the output scalar value *z*_*i*_(*k*) is calculated. Then, the error is evaluated as the difference $z_{i}\left(k\right)-S_{i}^{\prime }\left(k+1\right)$. The training error for node *i* is expressed over all *L-1* training pairs as ${\mathrm{Err}}_{i}=y_{i}-{\tilde{z}}_{i}$, where $y_{i}=\left(S_{i}^{\prime }\left(2\right),\ldots ,S_{i}^{\prime }\left(L\right)\right)$ and ${\tilde{z}}_{i}=\left(z_{i}\left(1\right),\cdots ,z_{i}\left(L-1\right)\right)$. This error is used to drive the optimization procedure.

After a number of epochs, the training is completed for the current node, and this process is repeated for all nodes of the VARDNN system. Currently, the VARDNN framework was implemented in MATLAB using the Deep Learning Toolbox version 12.1. Training was optimized using Adam (Kingma and Ba, 2014) with L2 regularization for the hidden layers to avoid over-fitting.

### Vector Auto-Regressive Deep Neural Network-Granger Causality Measure

*Granger causality* is a well-established method for the causal analysis of time series data, and the multivariate version (mvGC) has successfully been used to analyze fMRI BOLD signals (Goebel et al., 2003; Menon and Uddin, 2010; Marinazzo et al., 2011; Wen et al., 2012). The traditional approach to multivariate Granger causality uses a vector auto-regressive model involving a state vector ***S***(*t*), time lag *p*, coefficient vector ***A***_*ik*_, and residual *ε*_*i*_(*t*). The state of node *i* can be described by the following model:


(4) $$\begin{array}{r} S_{i}\left(t\right)=\sum_{k=1}^{p}{\text{A}}_{ik}{\text{S}\left(t-k\right)}^{T}+\varepsilon_{i}\left(t\right). \end{array}$$

Removing the past dependency of node *i* on *j*, the model can be written as


(5) $$\begin{array}{r} S_{i\setminus j}\left(t\right)=\sum_{k=1}^{p}{\text{A}}_{ik\setminus j}{{\text{S}}_{i\setminus j}\left(t-k\right)}^{T}+\varepsilon_{i\setminus j}\left(t\right). \end{array}$$

Then the mvGC causal index is expressed as


(6) $${ℱ}_{j\to i}=\log\left(var\left(\varepsilon_{i\backslash \text{j}}\right)/var\left(\varepsilon_{i}\right)\right)$$

where ***ε***_*i*_ and ***ε***_*i*\j_ are time series of residuals, and *var*() denotes the statistical variance. For mvGC, the coefficients and residuals of the vector auto-regressive model are calculated by OLS. However, as mentioned in section “Introduction,” neural network-based measures of GC have recently been proposed. VARDNN can also calculate this type of Granger causality. We simply use ${\mathrm{Err}}_{i}=y_{i}-{\tilde{z}}_{i}$ of a single node *i* as the residuals ***ε***_*i*_ and the error when removing the signal generated from node *j* from the optimization, ${\mathrm{Err}}_{i\backslash \text{j}}=y_{i}-{\tilde{z}}_{i\backslash \text{j}}$ as the residuals ***ε***_*i*\j_. Then, the VARDNN-GC measure is defined as


(7) $${ℱ}_{j\to i}^{VARDNN-GC}=\log\left(var\left({\mathrm{Err}}_{i\backslash \text{j}}\right)/var\left({\mathrm{Err}}_{i}\right)\right)$$

In the context of neuroscience, the process of removing the signal from another node can be thought of as performing a simulated lesion (Honey et al., 2007; Alstott et al., 2009). This virtual impairment can allow us to understand the effect of possible lesions in terms of their (Granger causal) connectivity relationships. Therefore, VARDNN can also function as a lesion model.

### Vector Auto-Regressive Deep Neural Network-Directional Influence Measure

Vector auto-regression is a well-known statistical approach to model the relationship between a scalar response and one or more explanatory variables. From this perspective, we can describe the state of a node *i* as


(8) $$\begin{array}{c} S_{i}\left(t\right)=\alpha_{i,0}+\alpha_{i}S{\left(t-1\right)}^{\text{T}}+\varepsilon_{i}\left(t\right)\\ \;=\alpha_{i,0}+\sum_{k=1}^{n}\alpha_{i,k}S_{k}\left(t-1\right)+\varepsilon_{i}\left(t\right) \end{array}$$

with state vector ***S***(*t*), linear relationship vector ***α***_*i*_=(α_*i*,1_,⋯,α_*i*,*n*_) and a residual term *ε*_*i*_ (*t*). Now, if we set all inputs into the node as 1, in other words ***S***(*t*−1)=(1,⋯,1) we get


(9) $$S_{i}^{\text{one}}=\sum_{k=0}^{n}\alpha_{i,k}+\varepsilon_{i}\left(t\right)$$

If input to node j is set to 0, $S_{i\backslash \text{j}}^{\text{one}}$ is expressed as


(10) $$S_{i\backslash \text{j}}^{\text{one}}=\sum_{k\neq j}\alpha_{i,k}+\varepsilon_{i}\left(t\right)$$

Then, the magnitude of the linear relationship, α_*i*,*j*_, or *directional influence* (DI), from node *j* to *i* is expressed as


(11) $$\alpha_{i,j}=S_{i}^{\text{one}}-S_{i\backslash \text{j}}^{\text{one}}$$

The VARDNN formula is complex, but non-linearly extended from this VAR formulation. After training the VARDNN, the deep neural network weights of each node encode its non-linear relationships with all other nodes. In a similar manner to the linear case, we defined a new measure of *directional influence*, VARDNN-DI, that extracts the non-linear magnitude of causal relationships from the network weights. Here, the DI from node *j* to node *i* is defined as


(12) $${ℱ}_{j\to i}^{VARDNN-DI}=\left|z_{i}^{\text{one}}-z_{i\backslash \text{j}}^{\text{one}}\right|.$$

In this case, $z_{i}^{\text{one}}$ is calculated by setting all inputs into the node to 1, that is ***S***(*t*) = (1, ⋯, 1) and ***I***(*t*) = (1, ⋯, 1), and passing these values through its DNN. $z_{i\backslash \text{j}}^{\text{one}}$ is also calculated in the same way but with the connection weight from node *j* set zeros in the first hidden layer neurons of the DNN of node *i*. As a result, the learned non-linear relationship from node *j* to node *i* can be extracted.

### Pre-processing of Vector Auto-Regressive Deep Neural Network Teacher Data

Although it is not a restriction, the VARDNN was designed to preferably deal with data in the range [0, 1]. In practice it may occur that the teacher signals are not normalized to this range. For example, resting state fMRI (rsfMRI) BOLD data has a normal distribution centered around zero (Chen et al., 2003). Thus, we need to transform experimentally derived time series data into the [0, 1] range. To do this, the mean and standard deviation of the data are first estimated:


(13) $$\begin{array}{r} \mu^{{}^{*}}=\frac{1}{nL}\sum_{i=1}^{n}\sum_{t=1}^{L}S_{i}\left(t\right), \end{array}$$

(14) σ*=1nL∑i=1n∑t=1L(Si(t)-μ*)2.

The values of a particular time series, mapping to a node *i* are normalized by


(15) θi(t)=(Si(t)-μ*)/σ*.

Then, a sigmoid function is used to transform the signal into the [0, 1] range:


(16) $$S_{i}^{{}^{\prime }}\left(t\right)=1/\left(1+e^{-\alpha \theta_{i}\left(t\right)}\right),$$

where the coefficient α = 1 was used for our current experiments. This transformation was chosen as it deals with potential signal outliers (that, for example, can appear in fMRI BOLD data) better than a simple linear transformation (see Supplementary Figure 2 for an evaluation of the effect of sigmoid transformation). Finally, the resulting set of time series, $S_{i}^{\prime }=\left(S_{i}^{\prime }\left(1\right),\cdots ,S_{i}^{\prime }\left(L\right)\right)$, is used as teaching data for setting the node states of the VARDNN.

### Performance Evaluation

We quantified the performance of the VARDNN-GC and VARDNN-DI measures over four different evaluations. The first evaluation confirmed the ability of VARDNN to learn and reproduce time series generated from uniformly distributed random numbers. The second evaluation estimated the ground truth directed graph from synthetic fMRI BOLD signals generated by the DCM, in order to clarify the relationship between network density and estimation accuracy. The third evaluation estimated the ground truth directed graph from synthetic neural activity signals generated by the reduced Wong-Wang model. This was done to clarify the relationship between node number and estimation accuracy. The DCM and reduced Wong-Wang models were chosen to generate biologically plausible synthetic neural signals for evaluation. The final evaluation was to measure VARDNN’s performance on experimental data. Here, we evaluated Alzheimer’s disease group separation ability using empirical rsfMRI BOLD signals. For each evaluation, we also compared our approach to other well-known functional connectome analysis algorithms, listed in Table 1. Of these algorithms, two are *zero-lag*-based algorithms: FC and PC. FC is the simplest measure of *pairwise* similarity between two time series, based on covariance. We used the corr() function from MATLAB (R2019b) for the comparison. PC is a conditional version of FC. It refers to the normalized correlation between two time series. We used the partialcorr() function of MATLAB to calculate the PC. Because PC relies on linear regression, it may face large dimensionality and multi-collinearity problems. Therefore, we implemented several countermeasure combined versions of PC, namely Principal Component PC (PC-PC), Elastic Net PC (EN-PC), and Partial Least Squares PC (PLS-PC). Elastic Net requires hyperparameters α and λ; we calculated these values from the minimum error of fivefold cross-validation using half of the sample data.

**TABLE 1:** Comparison algorithms.

Name	Abbreviation	Type	Strategy	Implementation
Functional connectivity (Correlation)	FC	Zero-lag	Pairwise	MATLAB function
Partial correlation	PC	Zero-lag	Multivariate	MATLAB function
Principal component + Partial correlation	PC-PC	Zero-lag	Multivariate	Our Script (MATLAB)
Elastic net + Partial correlation	EN-PC	Zero-lag	Multivariate	Our Script (MATLAB)
Partial least square + Partial correlation	PLS-PC	Zero-lag	Multivariate	Our Script (MATLAB)
Pairwise Granger causality	pwGC	Predictive	Pairwise	Our Script (MATLAB)
Multivariate Granger causality	mvGC	Predictive	Multivariate	Our Script (MATLAB)
Principal component + Multivariate Granger causality	mPC-GC	Predictive	Multivariate	Our Script (MATLAB)
Elastic Net + Multivariate Granger causality	mEN-GC	Predictive	Multivariate	Our Script (MATLAB)
Partial Least Square + Multivariate Granger causality	mPLS-GC	Predictive	Multivariate	Our Script (MATLAB)
Partial Conditioning Granger causality	PCGC	Predictive	Multivariate	Script (MATLAB)
Recurrent neural network Granger causality	RNN-GC	Predictive	Multivariate	Script (Python)
Linear estimator-uniform embedding (Transfer entropy)	LINUE-TE	Predictive	Multivariate	MuTE (MATLAB)
Nearest neighbor estimator-Non-uniform embedding (Transfer Entropy)	NNNUE-TE	Predictive	Multivariate	MuTE (MATLAB)

The *pairwise* GC and *multivariate* (or conditional) GC algorithms were implemented as MATLAB scripts. The GC time lag *p* parameter was empirically chosen as 3 for the evaluation. Multivariate GC, implemented as a multivariate vector auto-regression (mVAR) model, attempts to detect causal effects by considering differences between the network with all nodes and the network with the removal of a single node. On the other hand, the classical pairwise model simply considers two-node interactions. The mVAR model is widely used in many different fields, but it is also expected to suffer from the problems of data with large dimensionality and multicollinearity—the same as for the PC case. Therefore, we also implemented several counter-measure combined mvGC versions, namely multivariate Principal Component PC (mPC-GC), multivariate Elastic Net GC (mEN-GC), and multivariate Partial Least Squares GC (mPLS-GC) for our comparison.

Partially Conditioned GC (PCGC) is Granger causality based on partial conditioning, proposed by Marinazzo et al. (2012). The MATLAB script of its implementation was obtained from the GitHub.^1^ Parameter “ndmax” of the init_partial_conditioning_par_m() function was calculated based on node number, and the partial_CGC_fix_nd_m() function was used to acquire the dFC matrix.

The RNN-GC implementation was obtained from the GitHub.^2^ The evaluation environment was built using Anaconda3,^3^ Python-3.6, Keras-1.2.2, and Tensorflow-1.14.0.^4^ The dFC matrix was estimated as the average of 8 runs of the RNN_GC() function. In the first evaluation, we chose 30 as the number of hidden neurons.

Transfer entropy (TE) is a powerful tool for detecting the transfer of information between nodes and is capable of discovering non-linear interactions. It has been proven that TE is equivalent to Granger causality for data that can be assumed to be drawn from a Gaussian distribution (Barnett et al., 2009). We used the MuTE implementation (Montalto et al., 2014) in MATLAB for our comparison. This implementation automatically chooses the time lag based on the Bayesian information criterion. MuTE has three entropy estimators: the linear estimator (LIN), binning estimator (BIN), and nearest neighbor estimator (NN). Either a uniform or non-uniform embedding scheme is used to derive the transfer of information. As per the author’s results, BIN-NUE shows good estimation in both the linear and non-linear cases, but our initial tests found that estimating a dFC matrix from the signals generated by DCM was unsuccessful. Therefore, we chose the LIN-UE and NN-NUE algorithms for our comparison.

In sections “VARDNN Performance With Respect to Network Density” and “VARDNN Performance With Respect to Node Count,” various measures were used to estimate the ground truth directed graph, and in order to quantify them, evaluation was performed by changing the acceptance threshold and drawing an ROC curve. The ground truth matrix and result matrix were compared across the maximum value of the result matrix to its minimum. From this changing threshold, true and false positive rates of the result matrix were detected, and a ROC curve was generated from (0,0) to (1,1). After completing the ROC curve an area under the curve (AUC) value could be obtained. An AUC value close to 1 means a better classifier, whereas an AUC value close to 0.5 means that it is a random classifier. In this way, the accuracy of each measure was quantified by the value of AUC and used for our evaluation.

### Synthetic Data for Algorithm Evaluation

#### Uniformly Distributed Random Data

In section “VARDNN Trained on Random Independent Signals,” 8-nodes (*i* = 1…8) of 100 length time series were generated by the Mersenne Twister (Matsumoto and Nishimura, 1998) pseudo random generator, with values in the range [0, 1] of the uniform distribution. The VARDNN was trained with these random signals to verify the DNN unit functionality of each node.

#### DCM Generated Data

In section “VARDNN Performance With Respect to Network Density,” we evaluated the relationship between network density and ground truth network estimation from signals generated by an 8-node DCM model. Eight nodes were chosen based on the confirmation that the VARDNN operated as expected based on the results given in section “VARDNN Trained on Random Independent Signals.” We designed 5 patterns of sparsely connected directed networks for generating synthetic fMRI BOLD signals with DCM. The network densities were 0.2, 0.25, 0.3, 0.41, and 0.5. Each DCM connectivity matrix ***A*** was derived from these network graphs. Each node had self-connections with a weight of 0.2, and inter-node connections had random weight values in the range [0.2, 0.5]. The DCM input connection matrix ***C*** was set to identity so that each node received endogenous fluctuations as random white noise to model the external environment. To generate synthetic fMRI BOLD signals, parameters of *T* = 300 (number of observations) and *TR* = 2 (repetition time) were used. Endogenous fluctuations for each node were generated by the spm_rand_mar() function, and fMRI BOLD signals were generated by the observer function spm_int_J() with spm_fx_fmri and spm_gx_fmri routines. In addition to the sparse network densities, we also evaluated networks with dense connectivity at the levels found in anatomical studies of the mammalian brain, for example 97% of all possible cortico-cortical connections have been observed in mouse anatomical results (Gămănuţ et al., 2018). Here, all nodes were connected (giving a network density of 1.14 when including self-connections), with uniform random weight values of [−0.1, 0.1] for designating weak connections, and weight values of [0.2, 0.5] for strong connections. Due to the DCM formulation, we found that such fully connected networks easily exhibit divergence, and signals could reach a plateau value. By setting negative weights on some of the weak connections, a stable signal could be produced. We designed 5 patterns of directed networks with strong weight (|*a*_*ij*_| > 0.2) network densities set to 0.05, 0.11, 0.16, 0.27, and 0.36. In total, 10 patterns of 8-node fMRI BOLD signals and exogenous signals were generated by DCM for the evaluation.

#### Reduced Wong-Wang Generated Data

In section “VARDNN Performance With Respect to Node Count,” we evaluated the relationship between network size and ground truth network estimation from synthetic neural activity signals generated by the reduced Wong-Wang model implemented in The Virtual Brain (TVB) platform. We generated global coupling matrices, defined as ***C***, for the reduced Wong-Wang model by down-sampling Allen’s mouse structural connectome matrix (Oh et al., 2014; Melozzi et al., 2017) that was constructed from a number of anterograde tracer injections. We chose the reduced Wong-Wang model along with structural connectome data to produce biologically plausible dynamics for analysis. We wanted to evaluate causal measures in a more biologically plausible setting; real structural connectivity data could act as a useful proxy for defining a realistic ground truth network for our analysis. Furthermore, the reduced Wong-Wang model was chosen to explore network size over the DCM model, as the latter was found to be too computationally expensive when a large number of nodes were used. Node counts were 16, 32, 48, 64, 80, and 98. Similar to the DCM case, all nodes were connected with weight values in [−0.05, 0.01], with a fraction of 0.15 connections assigned a strong weight value (|*c*_*ij*_| > 0.2). The TVB framework was run using Anaconda3 and Python-3.6. We made small modifications to TVB’s reduced Wong-Wang implementation to obtain appropriate signals for the evaluation. The “state_variable_boundaries” constant was bound to the range [0, 1], which could clamp activity signals, and we modified this value [−2.5, 2.5] in order to preserve causal relationships. Because the reduced Wong-Wang model has an internal noise factor, η_*i*_(*t*), we wanted to use this noise signal as an exogenous input for the VARDNN. Therefore, both the endogenous noise and neural activity signals were recorded and exported for evaluation. In summary, 6 patterns were used to generate 16–98 node sets of neural activity and internal noise signals. These were generated at a sampling frequency of 64 Hz, a global coupling *G* = 1 set to provide sufficient dynamics between regions, and a noise amplitude σ = 0.014.

### Empirical Data Used to Evaluate Vector Auto-Regressive Deep Neural Network Performance

#### Alzheimer’s Disease Neuroimaging Initiative Database

Data used in the preparation of this article were obtained from the *Alzheimer’s Disease Neuroimaging Initiative* (ADNI) database.^5^ The ADNI was launched in 2003 as a public-private partnership, led by Principal Investigator Michael W. Weiner, MD. The primary goal of ADNI has been to test whether serial magnetic resonance imaging (MRI), positron emission tomography (PET), other biological markers, and clinical and neuropsychological assessment can be combined to measure the progression of mild cognitive impairment (MCI) and early Alzheimer’s disease (AD). Table 2 shows the demographics of healthy controls (HC) and AD subjects from the *ADNI2* subset of the database. The number of subjects with HC and AD were 41 and 32, respectively. Younger subjects with AD were preferred; HC of similar ages were selected.

**TABLE 2:** Demographics of HC and AD subjects.

Variables	AD (*n* = 32)	HC (*n* = 41)	*p*-value
Male/Female	14/18	17/24	0.851
Mean Age (SD)	72.4 (2.2)	72.1 (2.6)	0.454

#### Resting State fMRI Data Pre-processing

fMRI BOLD data from the *ADNI2* was scanned using a 3.0 Tesla Philips MRI scanner and saved in the DICOM format. The experimental settings of the scanner were: flip angle = 80, repetition time (TR) = 3,000 ms, echo time (TE) = 30 ms, pixel size = 3.3 × 3.3 mm, slice thickness = 3.3 mm, matrix size = 64 × 64 × 48, and frame length = 140. More information on the resting state parameters can be found on the ADNI website.

We converted the DICOM data to 4D NIfTI files using the dcm2nii tool (Li et al., 2016). Images were re-orientated so that the anatomical axial, sagittal, and coronal planes appeared correctly. Next, the CONN toolbox (Whitfield-Gabrieli and Nieto-Castanon, 2012) was used to pre-process the fMRI data. CONN registered NII images to the standard MNI brain space. Slice-timing correction was 1, 3, 5, …, 2, 4, and 6 for the Philips 3T MRI. We removed the first 10 frames of fMRI data, and the remaining data was smoothed by a FWHM of 5 mm. After pre-processing, the fMRI data was segmented using the CONN default atlas (132 ROIs), which is a combination of the FSL Harvard-Oxford atlas cortical and subcortical areas and the AAL atlas cerebellar areas. Voxels in each ROI were averaged to give representative fMRI BOLD signals for 132 regions over 130 frames.

#### Subject-Wise Evaluation Framework for Algorithm Comparison

We analyzed fMRI BOLD signals of HCs and ADs using 12 algorithms in Table 1 except RNN-GC and NNNUE-TE to estimate connectivity between brain regions. This generated 132 × 132 sized functional connectome matrices for each of the 41 HC’s and 32 AD’s results. These two groups were compared using statistical testing. We performed the Lilliefors test to check the distribution of functional connectome matrices and the Wilcoxon rank sum test (Mann-Whitney *U*-test) to compare the two groups. From this comparison, we obtained a 132 × 132 *p*-value matrix describing the actual performance of lesion detection by each analysis algorithm. The top *N*_*rr*_ ( = 30–300 step by 30) most different regional relationships between the HC and AD (that is, the set with the *N*_*rr*_ smallest *p*-values in the matrix) were then used to separate subjects to HC or AD group If an input functional connectome value (for a particular regional relationship) generated from new subject data was within the range [−βσ, βσ] (where β = 1.5–2.0 with a stepsize of 0.1) of the AD’s functional connectome population distribution, it is counted as “unhealthy”; otherwise, it is counted as “healthy.” This was done for each of the input functional connectome values in the *N*_*rr*_ most different regional relationships and a “healthy count” was acquired. The “health rating” for a subject is simply derived by dividing the health count by *N*_*rr*_.


(17) $$\begin{array}{c} Healthrating\\ =\frac{Healthycount\left(ofN_{rr}regionalrelationships\right)}{N_{rr}} \end{array}$$

Two thresholds of *N*_*rr*_ and β were swept over for the subject-wise evaluation. From this the ROC curves and area under the curves (AUCs) of a total of 60 conditions were calculated to quantify and compare the performance of each of the studied analysis algorithms. Note that this subject-wise evaluation framework cannot be used as a reliable diagnostic technique if the subject number is not large enough—which we realize is the case for the dataset we used in this section. In such cases, cross-validation will show different *p*-value matrices and top *N*_*rr*_ regional relationships and give unstable diagnosis results. However, if there are enough subjects, we believe the presented statistical framework can be reliably applied for AD diagnosis.

### Statistical Analysis Methods

To test the obtained AUC values, we used Kruskal-Wallis ANOVA in MATLAB for significance testing, and the Steel test in R-4.0.3 for multiple comparisons. For normality testing of the functional connectome distribution, we used the Lilliefors test in MATLAB. To perform non-parametric testing between the HC and AD groups, we used the Wilcoxon rank sum test (Mann-Whitney *U*-test) in MATLAB. Statistical significance was set at *p* < 0.05.

## Results

### Vector Auto-Regressive Deep Neural Network Trained on Random Independent Signals

**FIGURE 2:**
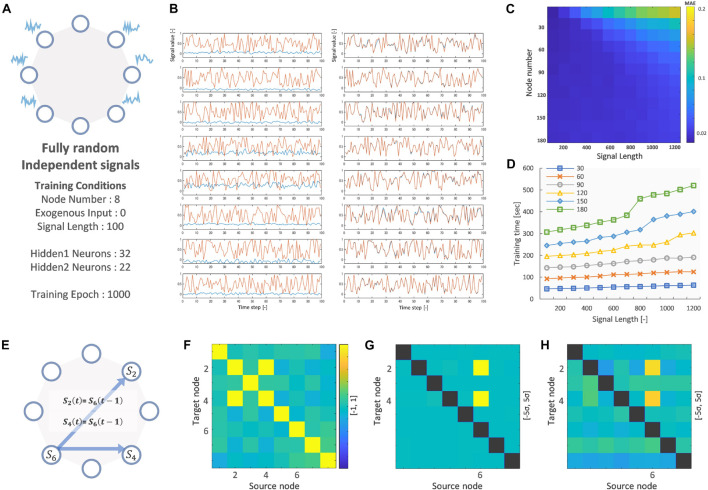
**(A)** Node network image and training conditions. **(B)** Left shows before training signals of 8 nodes (Red: teaching signal, Blue: VARDNN output). Right shows after training signals. **(C)** Training error result (MAE) of signal length and node count. **(D)** Training time result of signal length and node count. **(E)** Node network image for evaluating FC, mvGC and VARDNN-GC. **(F)** FC result of E. Color range is [–1, 1]. **(G)** mvGC result of E. Color range is *[*–*5σ, 5σ].* **(H)** VARDNN-GC result of E. Color range is *[*–*5σ, 5σ].*

Figure 2 shows the result of training the VARDNN on fully random and independent signals for 8-nodes. The following hyperparameters were used: training epochs = 1,000, and hidden layer 1 neuron count “hidden1” = 32 and layer 2 count “hidden2” = 22. The number of neurons per layer were empirically calculated by the method described in Supplementary Figure 1. Before training, the VARDNN nodes output signals clearly different from the teacher data, but after training with the conditions depicted in Figure 2A, the VARDNN nodes were able to output signals similar to the teaching signal (Figure 2). Before training, the mean absolute error (MAE) was 0.456 and after training it became 0.018. Figures 2C,D show some performance measures for VARDNN training. The MAE and training time were measured while changing the number of nodes and signal length, with the number of training epochs = 1,000. For any node number the VARDNN converged well and MAE showed values less than 0.02. Training time took less than 1 min with 30 nodes and signals of length 100 frames, and around 9 min with 180 nodes and signals of length 1,000 frames on a computer with the following specifications: OS, Windows 10 Pro; CPU, AMD Ryzen Threadripper 3970X 32-Core Processor (3.70 GHz); Memory, 128 GB. This makes VARDNN training very practical for data needing 100 nodes or a similar order of magnitude (such is the case when considering the number of cortical regions defined in brain atlases). Next, we performed a simple check to confirm VARDNN’s ability to estimate dFC; node 6′s signal was copied to node 2 and 4 at the next time step. The VARDNN was trained with new network (Figure 2E). Zero-lag analysis (FC) detected a strong relationship between nodes 2 and 4 (Figure 2F). On the other hand, mvGC and VARDNN-GC detected strong causality from node 6 to nodes 2 and 4 (Figures 2G,H). Since Granger causality returns values that differ by two or more digits, the matrix is displayed as a deviation.

### Vector Auto-Regressive Deep Neural Network Performance With Respect to Network Density

**FIGURE 3:**
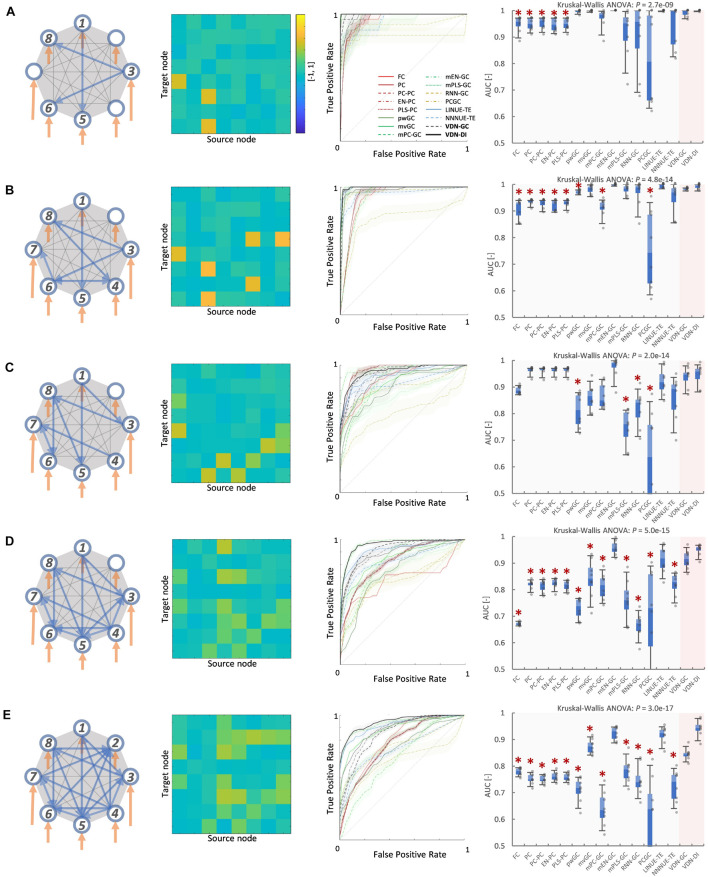
**(A)** Sparse pattern of 8-node network of network density 0.2. Eight self-connections and three other connections. From left to right images show the ground truth network graph, the matrix *A* for the DCM, the ROC curve result of ground truth estimation by each analysis algorithms, and the box-plot of AUC results, respectively. The color bar describes the matrix *A-*values for **(A–E)**. In the box-plot, a black dot shows each experimental result and a red asterisk shows significant difference from VARDNN-DI to other algorithms by Steel test (*p < 0.05) (*N* = 8). **(B)** Result of network density 0.25 (sparse pattern of 8-node network). **(C)** Result of network density 0.3. **(D)** Result of network density 0.41 **(E)** Result of network density 0.5.

**FIGURE 4:**
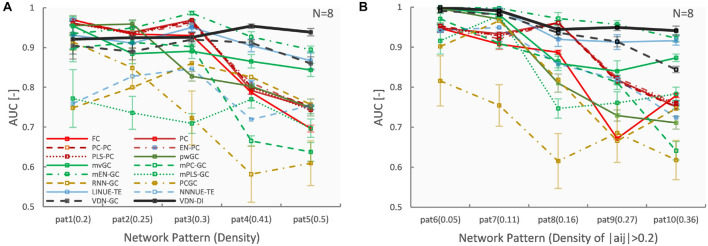
**(A)** Network density (sparse pattern) vs. AUC result. Error bar shows standard error. **(B)** Network density (fully connected) vs. AUC result.

**FIGURE 5:**
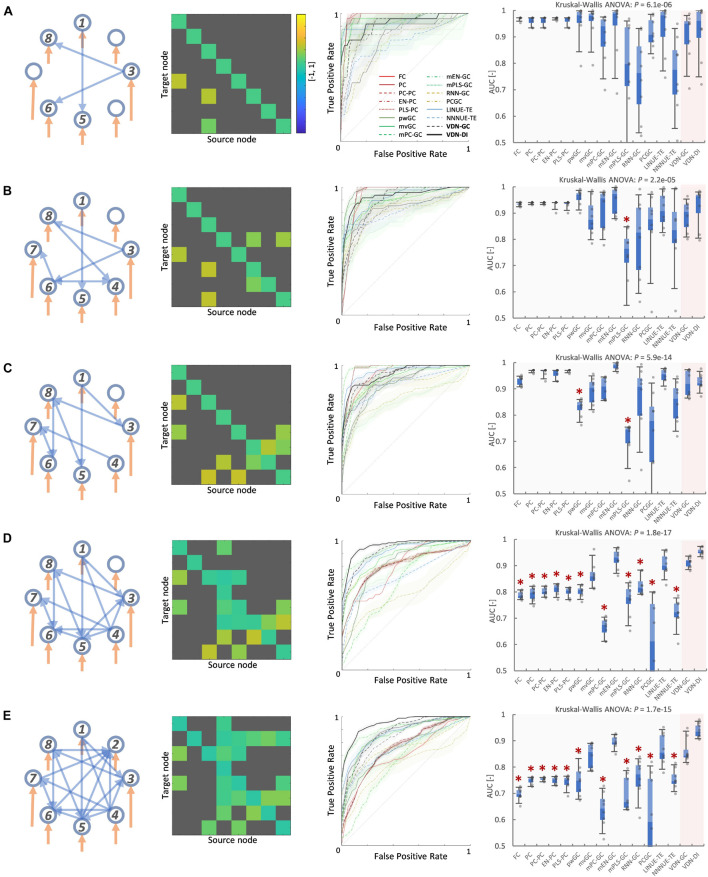
**(A)** Fully connected 8-node network with strong weight (| aij| > 0.2) network density 0.05. From left to right, images show the ground truth network graph, the matrix *A* for DCM, the ROC curve result of ground truth estimation by each analysis algorithm, and the box-plot of AUC results, respectively. The color bar describes the matrix *A-*values for **(A–E)**. In the box-plot, a black dot shows each experimental result and a red asterisk shows significant difference from VARDNN-DI to other algorithms by Steel test (*p < 0.05) (*N* = 8). **(B)** Result for the strong weight (| aij| > 0.2) network density 0.11 (fully connected 8-node network). **(C)** Result of strong weight network density 0.16. **(D)** Result of strong weight network density 0.27. **(E)** Result of strong weight network density 0.36.

Figures 3, 4 A show evaluation results for the relationship between sparse pattern network density and ground truth estimation from signals generated by the 8-node DCM. The signal length was 300, and the trial number was *N* = 8. The VARDNN was trained with the synthetic BOLD signals and exogenous signals with training epochs = 1,000, hidden1 neurons = 55, and hidden2 = 37 (determined by the method described in Supplementary Figure 1). Both VARDNN-DI and VARDNN-GC show competitive results against conventional algorithms. In particular, VARDNN-DI (AUC: 0.92–0.94 across densities) shows a significantly higher AUC score compared to other algorithms, or a similar AUC score to mEN-GC (0.99–0.89), LINUE-TE (0.93–0.87), and higher as the density of strong connections increased. The zero-lag analysis algorithms, FC and PC families, showed similar results in all cases. mPLS-GC, mPC-GC, PCGC, RNN-GC, and NNNUE-TE sometimes showed a high AUC score but appeared unstable. mEN-GC, LINUE-TE, and mvGC showed higher AUC scores relative to the others, except for the VARDNN-based algorithms (especially in the high network density case). Figures 4B, 5, show evaluation results for the relationship between strong weight (| a_*ij*_| > 0.2) fully connected network density and ground truth estimation. Once again, VARDNN-based algorithms also showed competitive results against existing algorithms. As in the sparse network evaluation, VARDNN-DI (AUC: 1.0–0.94) showed a significantly higher AUC score over most algorithms and a similar AUC score to mEN-GC (1.0–0.92), LINUE-TE (1.0–0.92), and became higher as the density of strong connections increased. Increased network density causes synergistic signal activation and multicollinearity problems and VARDNN demonstrated robust capabilities for dealing with these issues.

Computation time depends not only on the efficiency of the algorithm, but also on its implementation and runtime environment. However, as a basic guide we provide the computation times of the implementations we tested. The machine specification was the same as that described in section “VARDNN Trained on Random Independent Signals.” Many algorithms, such as FC, PC, mvGC, pwGC showed an order of about 10^–2^ s for 8 nodes. LINUE-TE (0.05 s) showed an order of about 10^–1^ s. VARDNN-based algorithms (24 s) and NNNUE-TE (8 s) showed an order of about 10 s. On the other hand, RNN-GC (599 s) showed an order of more than 10^2^ s. Because of this, RNN-GC was excluded from the evaluation in section “VARDNN Performance With Respect to Node Count” due to an excessively long calculation time in the large node case. In addition, NNNUE-TE was also excluded from the same evaluation due to a memory usage error.

### Vector Auto-Regressive Deep Neural Network Performance With Respect to Node Count

**FIGURE 6:**
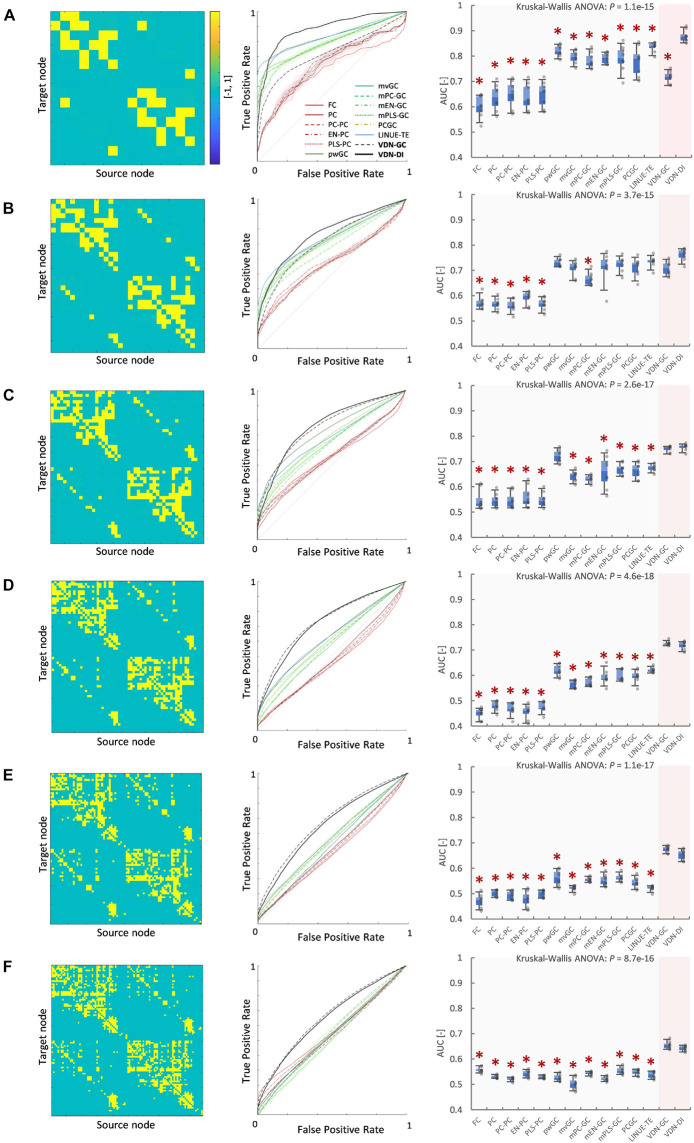
**(A)** Fully connected 16-node network with strong weight (| cij| > 1) network density 0.15. Node signals were generated by the reduced Wong-Wang model from the TVB software. From left to right images show the global coupling matrix *C* for the reduced Wong-Wang model, the ROC curve result of ground truth estimation by each analysis algorithm, and the box-plot of AUC results, respectively. Color bar shows matrix *C-*value for **(A–F)**. In the box-plot, black dot shows each experimental result and red asterisks denote a significant difference from VARDNN-DI to other algorithms by the Steel test (*p < 0.05) (*N* = 8). **(B–F)** Results for the 32-node, 48-node, 64-node, 80 node, and 98-node networks, respectively.

**FIGURE 7:**
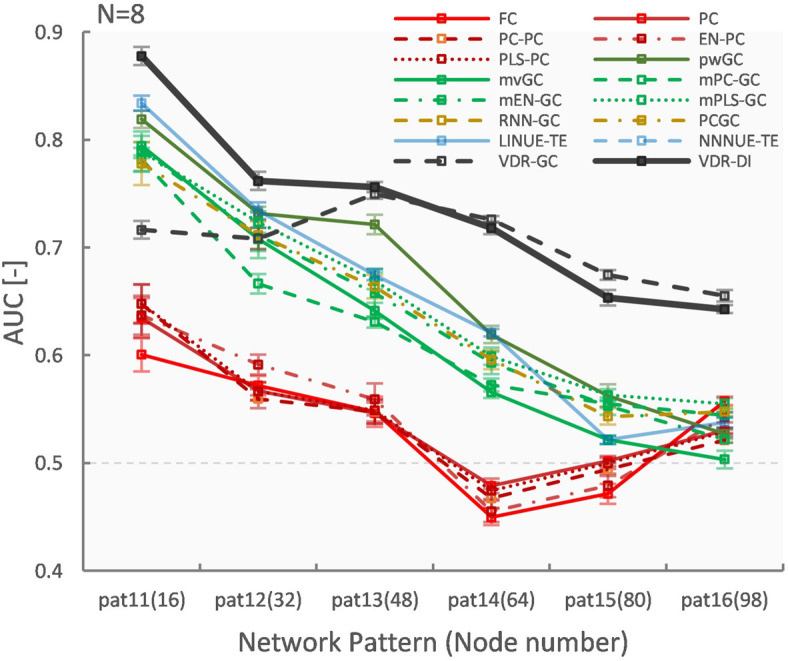
Node number (fully connected) vs. AUC result.

Figures 6, 7 show evaluation results for the relationship between node count and ground truth estimation of node signals generated by the reduced Wong-Wang model. Conventional algorithms such as multivariate GC (AUC: 0.79–0.5), pairwise GC (AUC: 0.82–0.53), and LINUE-TE (AUC: 0.83–0.54) struggled when the number of nodes was large. As they are based on OLS, their sensitivity is reduced by the number of free parameters. Countermeasure combined mvGC showed a small advantage over standard mvGC. On the other hand, VARDNN-DI (AUC: 0.88–0.64) and VARDNN-GC (AUC: 0.74–0.66) showed better scaling performance. At node numbers above 48, VARDNN-DI and VARDNN-GC showed significantly higher AUC scores over LINUE-TE and the mvGC family, and above 64 was significantly higher than pwGC. The pwGC algorithm showed better scores than mvGC; this may be due to the number of free parameters. VARDNN-GC did not perform well for the smallest network size, but unlike the rankings in section “VARDNN Performance With Respect to Network Density,” it outperformed VARDNN-DI for more than 64 nodes. Many zero-lag analysis methods did not perform well in any node count. This was because the sampling frequency (64 Hz) was too fast and correlations between nodes were lower than the fMRI BOLD signal repeat time (0.5 Hz). Overall, VARDNN showed a better AUC over linear regression results. This means VARDNN has the ability to be robust to redundancy or dealing with large dimensionality data. Interestingly, VARDNN shows a higher AUC over countermeasure techniques. This difference may be due to VARDNN’s non-linearity.

### Vector Auto-Regressive Deep Neural Network-Directional Influence and -Granger Causality Can Successfully Analyze Alzheimer’s Disease

**FIGURE 8:**
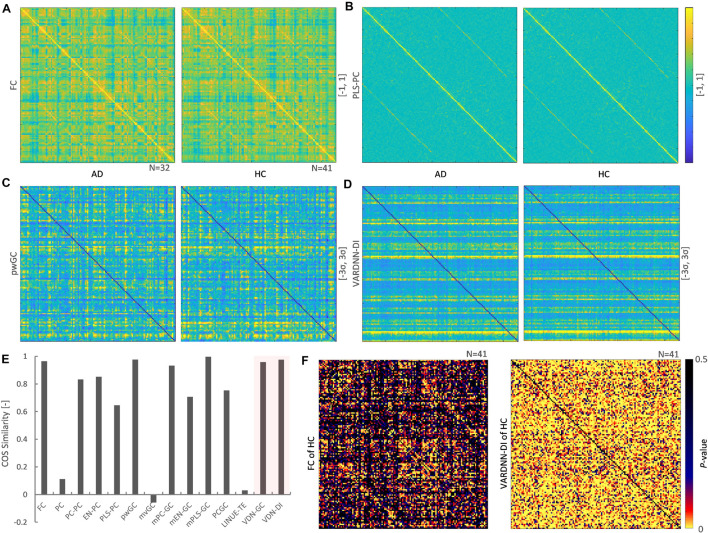
**(A)** Left: mean FC matrix (132 × 132) of the AD group (*N* = 32). Right: mean FC matrix of the HC group (*N* = 41) Color range is [–1, 1]. Source is column, target is row. **(B)** Mean PLS-PC matrix (left: AD group, right: HC group). Color range is [–1, 1] **(C)** Mean pwGC matrix (left: AD group, right: HC group). Color range is [–3σ, 3σ] **(D)** Mean VARDNN-DI matrix (left: AD group, right: HC group). Color range is [–3σ, 3σ] **(E)** Cosine similarity of the mean matrix between the AD and HC group matrices. **(F)** Lilliefors test result matrix of FC (left) and VARDNN-DI (right) of HC.

The rsfMRI data acquired from *ADNI2* was preprocessed then separated by the CONN default atlas, generating 132 regions and 130 frames of rsfMRI BOLD signals. These were analyzed by several analysis algorithms to obtain functional connectome matrices. VARDNN was trained with the empirical BOLD signals and randomly generated exogenous signals (uniform distribution in range [0, 1], acting as a noise factor to the algorithm), with training epochs = 1,000, hidden1 neurons = 34, and hidden2 = 23 (calculated by the method described in Supplementary Figure 1). Figures 8A–D show the mean functional connectome matrices for the AD and HC subjects. Most algorithms showed similar patterns for AD and HC, but PC, mvGC, and LINUE-TE did not (Figure 8E). The cosine similarities between AD and HC matrices were FC (0.965), PLS-PC (0.645), pwGC (0.976), VARDNN-DI (0.976), PC (0.11), mvGC (−0.025), and LINUE-TE (0.034). This meant that FC, pwGC, VARDNN, countermeasure combined PC and mvGC consistently captured meaningful regional relationships across the AD and HC subjects, but PC, mvGC, and LINUE-TE were clearly insensitive because of the large dimensionality problem.

To compare the values of functional connectome matrices (132 × 132) between the AD and HC groups we needed to test the normality of their distributions. FC values in the matrices for the HC group (21.3%) were significantly *not* Gaussian distributed (*p* < 0.05) (Figure 8F, left). VARDNN-DI values in the matrices for the HC group (74.1%) were significantly *not* Gaussian distributed (*p* < 0.05) (Figure 8H right). From these results, a non-parametric test was chosen as appropriate for comparing the AD and HC groups. We used the Wilcoxon rank sum test (Mann-Whitney *U*-test) for this comparison.

**FIGURE 9:**
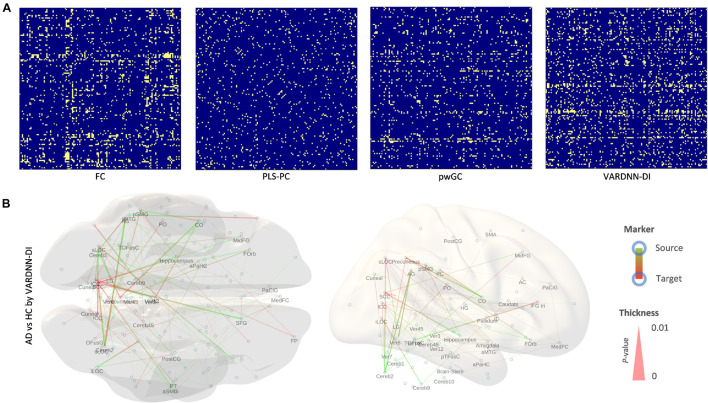
**(A)** Significantly different (*p* < 0.05, yellow color; non-significant, blue color) regional relationship matrices between AD and HC groups. From left to right, FC, PLS-PC, pwGC, and VARDNN-DI, respectively. **(B)** The whole brain image of the most different (0 ≤ *p* < 0.0024) regional relationships by VARDNN-DI. The image on the left is seen from the dorsal view and the right image shows the medial view of the left hemisphere. Posterior is left and anterior is right. Blue circles are region centroids. The source to target areas are color coded from green to red. A smaller *p*-value shows a thicker line.

Figure 9 shows comparison results between the AD and HC groups for each analysis algorithm. VARDNN-DI detected that 12% of regional relationships were significantly affected (*p* < 0.05) by Alzheimer’s disease; FC (10.5%), PLS-PC (5.5%), and pwGC (7.3%) were also able to detect a difference well. Even though these algorithms had functional connectome matrices showing high cosine similarity between AD and HC (Figure 8E), they could capture significant differences between the AD and HC groups. Next, we confirmed the consistency of our results with previous studies. Table 3 shows the top 20 most different regions (in terms of dFC) between the AD and HC groups, calculated from VARDNN-DI.^6^ Significantly different dFC were counted by brain region, and the total number of both sources and targets is displayed in the VDNdiTotal column in descending order. Table 3 columns are sorted by the bold values in the VDNdiTotal column. In this result, VARDNN-DI was able to detect lesion effects of the Default Mode Network (DMN), including precuneus cortex, angular gyrus, and cingulate gyrus (posterior division). Additionally, supramarginal gyrus (posterior division) and intracalcarine cortex were also detected and matched well with previous studies. This result confirms that whole-brain screening with VARDNN-DI may be useful for further brain analysis. Both FC and pwGC detected some common effects within the DMN. Although these causal and zero-lag analyses use different approaches, they were capable of showing similar results.

**TABLE 3:** Top 20 regions of most different dFC between AD and HC, calculated from VARDNN-DI.

ROI name	Network	FC	PC-PC	PLS-PC	pGCSrc	pGCTrg	VDNdiSrc	VDNdiTrg	VDNdiTotal	References
Precuneous (Precuneous cortex)	pDMN	20	14	8	11	18	30	59	**89**	Bakkour et al., 2013; Peraza et al., 2014; Utevsky et al., 2014; Zott et al., 2018; Pascoal et al., 2019
pSMG l (Supramarginal gyrus, posterior division left)	FPN/Lang (L)	24	13	5	12	4	37	43	**80**	Harasty et al., 1999; Bakkour et al., 2013; Peraza et al., 2014
ICC l (Intracalcarine cortex left)	Visual.Primary	6	12	9	10	4	19	58	**77**	Peraza et al., 2014; Utevsky et al., 2014; Hafkemeijer et al., 2015; Schwab et al., 2020
SCC l (Supracalcarine cortex left)	Visual.Primary	14	8	13	10	12	10	66	**76**	Utevsky et al., 2014; Hafkemeijer et al., 2015
AG l (Angular gyrus left)	lDMN	13	16	6	8	26	18	58	**76**	Bakkour et al., 2013
PC (Cingulate gyrus, posterior division)	pDMN	5	9	8	19	10	35	24	**59**	Bakkour et al., 2013
toITG l (Inferior temporal gyrus, temporooccipital part left)	DAN	17	9	6	8	1	18	41	**59**	Mosconi et al., 2009; Bakkour et al., 2013; Peraza et al., 2014; Utevsky et al., 2014; Zott et al., 2018; Pascoal et al., 2019
CO l (Central opercular cortex left)	Auditory	9	9	5	6	7	35	23	**58**	
Cuneal r (Cuneal cortex right)	Visual.Primary	6	9	7	9	3	14	41	**55**	Utevsky et al., 2014; Hafkemeijer et al., 2015
Cereb6 l (Cerebelum 6 left)	Cerebellum	10	2	7	15	15	33	19	**52**	Jacobs et al., 2018
SFG r (Superior frontal gyrus right)	FPN (R)	22	15	12	12	11	37	14	**51**	Bakkour et al., 2013
Pallidum l	Salience	13	9	7	16	8	18	32	**50**	Bakkour et al., 2013
SPL r (Superior parietal lobule right)	DAN	36	12	5	12	6	34	16	**50**	Peraza et al., 2014
Cereb9 l (Cerebelum 9 left)	Cerebellum/pPaHC	10	11	7	7	8	42	7	**49**	Jacobs et al., 2018
FOrb l (Frontal orbital cortex left)	Inferior Temporal	3	7	4	4	13	37	12	**49**	
Caudate r	Thalamus	16	6	8	5	35	28	20	**48**	
AG r (Angular gyrus right)	lDMN	15	3	9	10	0	23	24	**47**	
Cereb45 l (Cerebelum 4 5 Left)	Cerebellum	9	10	5	12	22	16	31	**47**	Peraza et al., 2014
sLOC l (Lateral occipital cortex, superior division Left)	sLOC	17	4	5	4	19	28	19	**47**	Bakkour et al., 2013
Ver12 (Vermis 1 2)	Cerebellum/pPaHC	34	9	6	7	3	19	27	**46**	

*From left to right column: ROI name, brain network name, significantly different region counted by FC, PC-PC, PLS-PC, pwGC source count, pwGC target count, VARDNN-DI source count, VARDNN-DI target count, total of VDNdiSrc and VDNdiTrg column, references, respectively.*

**FIGURE 10:**
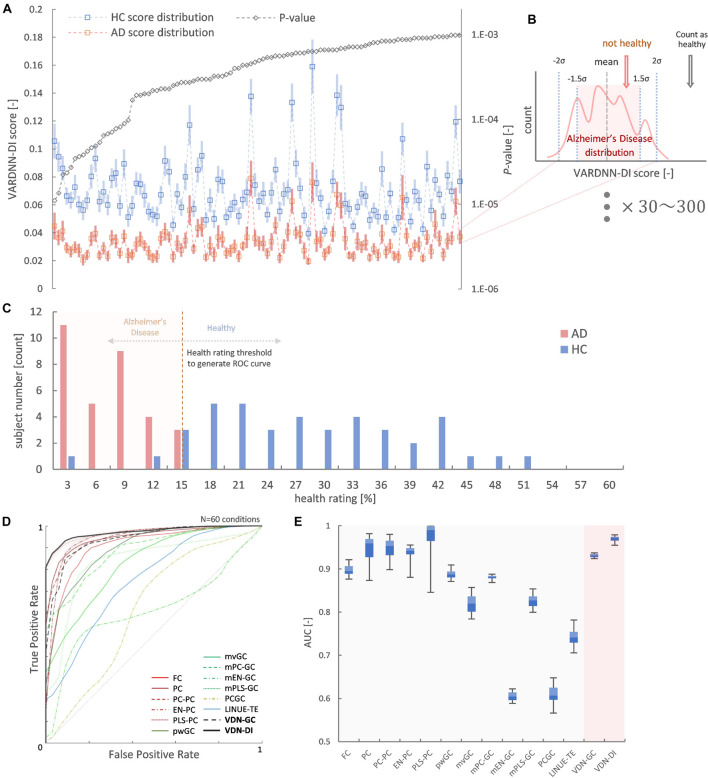
**(A)** Top 100 most different regional relationships by VARDNN-DI. The left vertical axis shows VARDNN-DI score and blue (HC) and orange (AD) lines show mean score (box) and standard error (bar). The right vertical axis shows the logarithmic scale of *p*-value. The black dotted line shows the *p*-value of the regional relationship between AD and HC groups. **(B)** Healthy count estimation for subject-wise evaluation framework between AD vs. HC. If the input VARDNN-DI score is within the specific σ range (1.5–2.0) of the AD distribution, it is counted as unhealthy. This operation was repeated from top *N*_*rr*_ (30–300) most different regional relationships. **(C)** Example histogram (*N*_*rr*_ = 100, β = 2, VARDNN-DI) of *health rating*. Red bars show AD subjects. Blue bars show HC subjects. **(D)** Mean ROC curves of 60 conditions by each analysis algorithm. **(E)** AUC results from **(D)**.

Figure 10 shows the result of Alzheimer’s disease separation ability by our subject-wise evaluation framework. We tried to use two subject groups as a ground truth to quantify the separation ability of each algorithm. Figure 10A shows the top *N*_*rr*_ ( = 100) most different regional relationships between the AD and HC groups by VARDNN-DI. From left to right, regional relationships were sorted in ascending order of *p*-value. As shown in this graph, the dFC distribution of AD was significantly different from that of HC. This means that connectivity is reduced in these regions. Then, we were able to use these *N*_*rr*_ regional relationships to separate AD and HC subjects. The functional connectome matrix was calculated from the rsfMRI BOLD signal obtained from a new subject, and the top *N*_*rr*_ regional relationships from the matrix were used to obtain their *health rating* (Figure 10B), which could be used to distinguish between AD (*N* = 32) and HC subjects (*N* = 41) (Figure 10C). ROC curves were generated from the subject distribution of AD and HC based on the *health rating* histogram. A “healthy” threshold to classify healthy or unhealthy was moved from 100 to 0% over the *health rating* range. In total, 60 conditions of the framework settings (*N*_*rr*_:30–300 with step-size 30, β threshold: 1.5–2.0 with step-size 0.1) were tested for each algorithm. Figure 10D shows ROC curve results for each analysis algorithm, and Figure 10E shows the box-whisker plot of AUCs. VARDNN-DI (0.97) showed the highest AUC and PLS-PC (0.969) showed a competitive AUC result. These algorithms separated the AD and HC groups well. FC (0.897) and pwGC (0.888) (both pairwise strategies) showed similar cosine similarities and subject-wise evaluation results in TR = 3 s rsfMRI BOLD data. They in turn showed a similar ability for group separation. The mvGC family of algorithms and LINUE-TE showed unstable subject-wise evaluation results—they did not separate the two groups well. The PC family showed high AUC scores, but they were unstable over several thresholds. Also, their cosine similarity showed unstable results, i.e., their similarities were smaller than other algorithms (Figure 8E) and significantly different regional relations were fewer (Figure 9). For many brain regions, the conditioning strategy shows very small differences around the zero value and the two group distributions were mixed. Even though some PC approaches were able to show good group separability, overall results were unstable. In summary, this demonstrates that VARDNN performs well with real-world, empirical rsfMRI BOLD data.

## Discussion

In this article, we presented VARDNN, a novel method for functional connectome estimation based on a vector auto-regressive deep neural network architecture. We proposed two VARDNN dFC measures that show good sensitivity to causal structures, namely VARDNN-GC and VARDNN-DI. In our experiments, the VARDNN was successfully trained with random uniformly distributed signals, synthetic fMRI BOLD signals that were generated by the DCM model, synthetic neural activity signals that were generated by the reduced Wong-Wang model, and empirical fMRI BOLD signals acquired from the *ADNI2* dataset. Functional connectome estimation of ground-truth synthetic fMRI BOLD signals from DCM was successful, and VARDNN-DI and VARDNN-GC showed competitive results with existing functional connectome estimation measures. In particular, VARDNN-DI showed a higher AUC score over other algorithms for the high network density case (Figure 5). Furthermore, functional connectome estimation from ground-truth synthetic neural activity signals generated by the reduced Wong–Wang model was evaluated. VARDNN-based dFC measures significantly outperformed other causal estimation measures for the large node count case (Figure 6). Several articles evaluating undirected or directed functional connectivity algorithms based on synthetic fMRI BOLD signals generated by DCM exist, for example, Smith et al. (2011) and Prando et al. (2020). However, the results of these experiments are difficult to compare due to their various network configurations and densities. For example, networks with node numbers of 5, 10, and 50 with very sparse connections were used in Smith et al. (2011), while node numbers of 7 and 66, with a network density 0.5 were used in Prando et al. (2020). In this work we evaluated both increasing network density and node number cases. The evaluation of multiple types of algorithms for the early diagnosis of Alzheimer’s disease has been performed by Dauwels et al. (2010). We designed a subject-wise evaluation framework to separate AD and HC groups from empirical rsfMRI BOLD data in order to quantify the performance of functional connectome estimation measures. In our results, VARDNN-DI especially succeeded in extracting lesioned brain regions consistent with previous studies (Table 3), and it showed a higher AUC score over existing functional connectome estimation measures when separating AD and HC groups (Figure 10). This result also supports the sensitivity of the VARDNN-DI measure.

Both multivariate Granger causality and LINUE transfer entropy rely on linear multivariate auto-regression. Thus, their sensitivity becomes low for large node counts and little sample data (Barnett and Seth, 2014). Several countermeasure techniques, such as PCA (Zhou et al., 2009), Elastic Net (Zou and Hastie, 2005; Ryali et al., 2012), and PLS (Wold et al., 2001; Krishnan et al., 2011) have been used to avoid these large dimensionality and multi-collinearity problems. We compared VARDNN against these techniques and VARDNN showed competitive or even better estimation results. If a DNN does not have (non-linear) activation functions, it is essentially the same as linear regression. Activation functions, such as ReLU, non-linearly extend the ability of linear regression. This strategy works well for extending the capability of GC by VARDNN. The well-studied training techniques of deep neural networks complements the weaknesses of linear regression and extends its capabilities.

We showed that VARDNN-based GC was able to obtain better results than RNN-GC, an established neural network-based approach. RNN-GC was directly compared with another neural network-based approach called NN-GC, which it outperformed (Montalto et al., 2015; Wang et al., 2018). The original NN-GC definition of causality was a simple subtraction: ***Err***_*reduced*_ − ***Err***_*full*_, and this definition showed less sensitivity than the RNN-GC definition of *log*⁡(*var*(***Err***_*reduced*_)/*var*(***Err***_*full*_)). Our VARDNN-GC measure used the same type of definition as RNN-GC and outperformed it. From the comparative results of Wang et al. (2018) we can also expect VARDNN to outperform NN-GC. RNNs require sequential input, which can make training convergence difficult. Our approach uses an auto-regressive DNN structure so mini-batch and shuffling during network training becomes possible. This flexibility improved the training time of the VARDNN and the accuracy of causality estimation. Furthermore, we defined a new dFC measure, VARDNN-DI, as |*z*_*full*_ − *z*_*reduced*_|, to extract causal relationships from trained DNN weights. This simple equation showed surprisingly high sensitivity in comparison to other algorithms. Owing to these properties, the VARDNN can be trained on non-linear signals and capture non-linear relationships in the weights of the DNNs.

Despite its effectiveness, there are some limitations to the VARDNN and its functional connectome measurements. Since the designs of the VARDNN-based dFC measures have similarities with *predictive*-based analysis, they have similar limitations to Granger causality (Bressler and Seth, 2011; Barnett and Seth, 2014; Stokes and Purdon, 2017). The first is variability in the shape and latency of Hemodynamic Response Functions (HRFs) in different brain regions (Miezin et al., 2000) and across subjects (Aguirre et al., 1998). Neural activity and HRFs in brain regions are not completely synchronized, so data driven functional connectome measures, including FC, PC, and GC, have always faced this issue. However, many biological experiments involve two group comparisons, for example AD vs. HC, wild type vs. mutated disease model, etc. In this scheme, the variability of HRFs in different brain regions and subjects can be considered as differentiating features between the two groups. Therefore, for such schemes, applying data driven functional connectome measures for rsfMRI BOLD signal analysis and striving to increasing the experimental number will support robustness of experimental results. The second issue is related to changes in the sampling time interval. In our experiments, VARDNN-DI detected lesioned brain regions from experimental rsfMRI BOLD signals with a repetition time (TR) of 3 s. These dFCs clearly depend on the repetition time. If the repetition time was changed to 1.5 s, the dFC results might have differed. This problem is not only for *predictive*-based analysis, but also for *zero-lag* analysis. Longer time intervals average over fine patterns in neural activity, and correlation-based analysis shows better sensitivity at lower as compared to higher sampling rates. Thus, we need to be careful and consider this time dependence when choosing the appropriate analysis algorithm. The third is with regard to negative causal relationships between nodes. The DCM inversion algorithm is able to estimate positive and negative connection weights, but GC-based algorithms basically do not have this function because they are calculated from changes in variance. Some GC approaches may generate negative values, but such values have no physical interpretation (Chen et al., 2006). This type of positive and negative causality estimation is also an issue for VARDNN.

We provide a VARDNN toolbox for MATLAB that implements the algorithms described in this paper. The code is open source and can be downloaded from the GitHub repository at https://github.com/takuto-okuno-riken/vardnn. The VARDNN toolbox contains a command line tool that includes several analysis algorithms, namely VARDNN-DI, VARDNN-GC, mvGC, pwGC, linear TE, FC, and PC to estimate a functional connectome from brain signals. We hope that this toolbox will help further brain analysis, and the toolbox will be expanded with new features in the future. The Deep Learning toolbox ver. 12.1 (or later), Fuzzy Logic Toolbox ver. 2.6 (or later), and MATLAB R2019a (or later) are required to run the VARDNN scripts.

## Data Availability Statement

The original contributions presented in the study are included in the article/Supplementary Material, further inquiries can be directed to the corresponding author/s.

## Ethics Statement

The studies involving human participants were reviewed and approved by the Alzheimer’s Disease Neuroimaging Initiative. The patients/participants provided their written informed consent to participate in this study.

## Author Contributions

TO contributed to conception and design of the study and wrote the first draft of the manuscript. Both authors contributed to manuscript revision, read, and approved the submitted version.

## Conflict of Interest

The authors declare that the research was conducted in the absence of any commercial or financial relationships that could be construed as a potential conflict of interest.

## Publisher’s Note

All claims expressed in this article are solely those of the authors and do not necessarily represent those of their affiliated organizations, or those of the publisher, the editors and the reviewers. Any product that may be evaluated in this article, or claim that may be made by its manufacturer, is not guaranteed or endorsed by the publisher.

## Footnotes

1
https://github.com/danielemarinazzo/PartiallyConditionedGrangerCausality

2
https://github.com/shaozhefeng/RNN-GC

3
https://www.anaconda.com/

4
https://www.tensorflow.org/

5
adni.loni.usc.edu

6 VARDNN-DI was chosen over VARDNN-GC as we found it gave better diagnosis results, implying that VARDNN-GC’s estimated significantly different regions were less accurate.
