# Supplementary material for: Vector Auto-Regressive Deep Neural Network: A Data-Driven Deep Learning-Based Directed Functional Connectivity Estimation Toolbox
Source: Front Neurosci. 2021 Nov 23;15:764796. doi: 10.3389/fnins.2021.764796 (PMC8651499; doi:10.3389/fnins.2021.764796)
Supplement: Supplementary file 1 [file Image_1.pdf]

Supplementary Material

1 Supplementary Figures

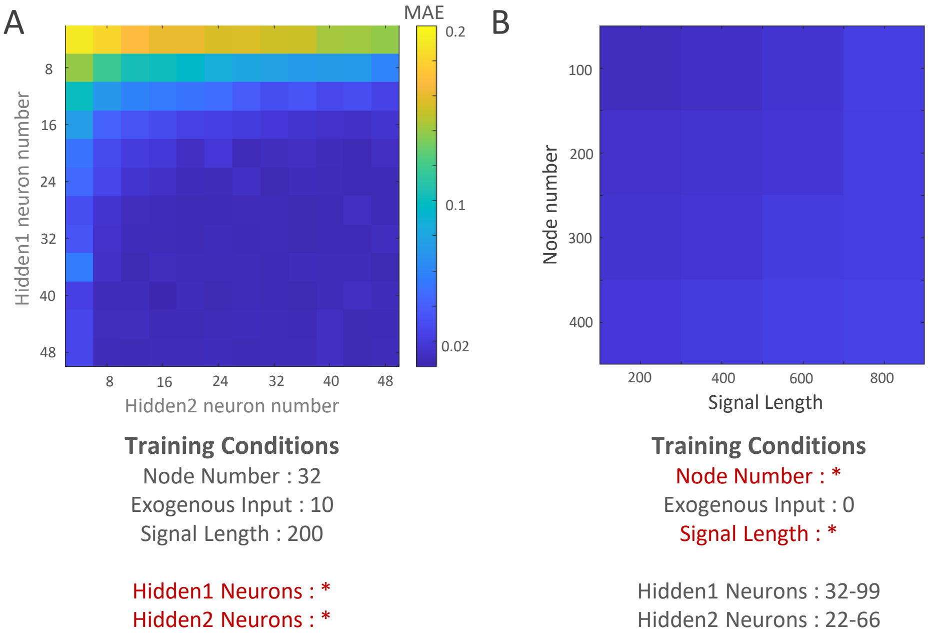

Supplementary Figure 1. A VARDNN node consists of a deep neural network unit. In our current implementation we used 3 layers: hidden layer 1, hidden layer 2 and an output layer (see Fig.1B). The last layer (output layer) contains only one neuron, and the number of neurons in hidden layer 1 (*hidden1*) and hidden layer 2 (*hidden2*) were empirically calculated by:

$$hidden1 = \left\lceil 32 + \frac{(SignalLength - 100) \times 0.12}{(1 + NodeCount \times 0.01)} \right\rceil \quad \text{(S.1)}$$

$$hidden2 = \left\lceil hidden1 \times \frac{2}{3} \right\rceil \quad \text{(S.2)}$$

Where *SignalLength* is the length of the time series data to train on, and *NodeCount* is the number of nodes to be used. (A) Mean Absolute Error (MAE) result for training with different combinations of hidden1 neurons vs hidden2 neurons. Training conditions were: node count=32 (independent nodes), exogenous input=10 and signal length=200 (random signals in the range [0, 1]). The form of Eq. S.2 was empirically determined. (B) MAE result of node number vs signal length with empirically determined *hidden1* and *hidden2* neurons based on Eq. S.1 and S.2. Results show a sufficiently small enough MAE with node numbers from 100 to 400 and signal lengths from 200 to 800.

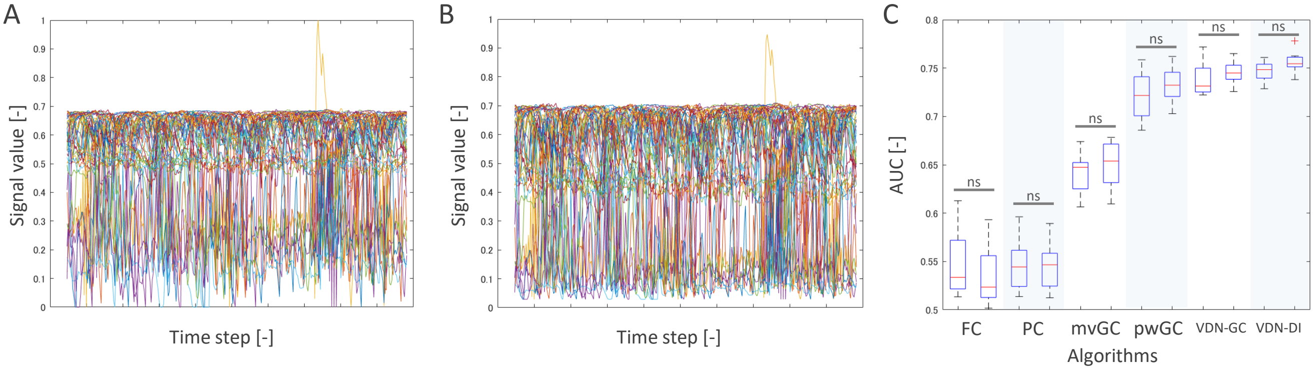

Supplementary Figure 2. (A) 48 node signals generated by the reduced Wong-Wang model from the TVB software. This is the same data as used for Fig.6C. (B) Sigmoid transformed signals (described in Section 2.4) (C) AUC comparison between original and sigmoid transformed signals for each tested algorithm (N=8, same data as used for Fig.6C). For each algorithm, the left box is the original and the right is transformed result. Some algorithms showed a small AUC improvement after sigmoid transformation, but no algorithm showed a statistically significant difference in results by the Mann-Whitney U test (ns $p \geq 0.05$).
